# Japanese Encephalitis Viruses from Bats in Yunnan, China

**DOI:** 10.3201/eid1506.081525

**Published:** 2009-06

**Authors:** Jing-Lin Wang, Xiao-Ling Pan, Hai-Lin Zhang, Shi-Hong Fu, Huan-Yu Wang, Qing Tang, Lin-Fa Wang, Guo-Dong Liang

**Affiliations:** Institute for Viral Disease Control and Prevention, Beijing, People’s Republic of China (J.-L. Wang, X.-L. Pan, S.-H. Fu, H-Y. Wang, Q. Tang, G.-D. Liang); Yunnan Institute of Endemic Disease Control and Prevention, Dali City, People’s Republic of China (J.-L. Wang, H.-L. Zhang); Australian Commonwealth Scientific and Industrial Research Organisation Australian Animal Health Laboratory, Geelong, Victoria, Australia (L.-F. Wang); 1 These authors contributed equally to this article.

**Keywords:** Japanese encephalitis, bats, epidemiology, phylogeny, China, viruses, zoonoses, dispatch

## Abstract

Genome sequencing and virulence studies of 2 Japanese encephalitis viruses (JEVs) from bats in Yunnan, China, showed a close relationship with JEVs isolated from mosquitoes and humans in the same region over 2 decades. These results indicate that bats may play a role in human Japanese encephalitis outbreaks in this region.

Bats have been increasingly recognized as an important source of zoonotic viruses responsible for some of the recent major disease outbreaks, including Hendra, Nipah, severe acute respiratory syndrome–associated, and Ebola viruses (*1*). *Japanese encephalitis virus* (JEV) is a member of the family *Flaviviridae* and the genus *Flavivirus* (*2*) and is the etiologic agent of severe encephalitic diseases in humans. In addition to humans, JEV has been isolated from various hosts, e.g., mosquitoes, birds, pigs, and horses (*3**,**4*). The role of bats in JEV epidemiology has been poorly defined, although the virus has been isolated from bats of various species since 1963 in multiple locations (*5**,**6*). To date, no nucleotide sequence information has been available for bat JEV isolates.

Yunnan Province, in southern China, has a tropical to subtropical climate and a diverse biota. The annual case rate of Japanese encephalitis in Yunnan is >2× the average case rate of the whole country (*7*). We report the molecular and virulence characterization of 2 bat JEV isolates from Yunnan: B58, obtained from a Leschenault’s rousette (*Rousettus leschenaultia*), a fruit bat, in 1989; and GB30, obtained from a little tube-nosed bat (*Murina aurata*), an insectivore, in 1997.

## The Study

The viruses used in this study were isolated from homogenates of the brains of bats by direct intracranial (i.c.) inoculation of 3-day-old suckling mice. Although isolate B58 was obtained in 1989, no further identification or characterization was conducted until this study. GB30 was identified as JEV by serologic analysis (*8*). Inoculation in suckling mice was conducted following procedures approved by the Animal Ethics Committee of the Institute for Viral Disease Control and Prevention, China. Mice were observed 2× per day after inoculation, and every 2 hours after the onset of clinical signs. After euthanasia, supernatant from brain homogenate was used to inoculate C6/36 cells. After the appearance of cytopathic effect (CPE), supernatant was harvested and passaged 3 more times. Virus stock was prepared from the previous passage and stored at –85^o^C.

Neurovirulence of the 2 bat JEV strains and of a mosquito-derived JEV strain, M10 (*9*), were determined. All viruses were consecutively passed 3 times in mice, and virus suspension (defined as the 10^–1^ stock) was prepared from the third passage. For determination of a 50% lethal dose (LD_50_), suckling mice (5 per group) were inoculated i.c. with dilutions from 10^–1^ to 10^–9^. Animals were monitored daily for survival, and the LD_50_ values were calculated by using a standard method (*10*).

Viral RNA was isolated by using the Viral RNA Mini Kit (QIAGEN, Hilden, Germany). First strand cDNA was synthesized using the Ready-To-Go kit (Amersham Pharmacia Biotech, Uppsala, Sweden). Flavivirus-specific primers (*11*) and primers designed from the sequence of JEV Beijing-1 (L48916) were used for PCR and sequencing. Sequence assembly was conducted by using the ATGC software package, version 4.0 (GENETYX Corp., Tokyo, Japan). Homology and alignment analysis were conducted by using ClustalX version 1.8 (www.clustal.org/download/1.X/ftp-igbmc.u-strasbg.fr/pub/ClustalX) and MegAlign (DNASTAR, Madison, WI, USA). MEGA 3.1 (*12*) was used for phylogenetic analysis.

For initial studies, suckling mice were inoculated i.c. with the supernatant of clarified brain homogenate. The 2 groups (n = 8 for each) inoculated with B58 and GB30, respectively, displayed clinical signs after 42 h postinoculation (hpi), whereas the control group with buffer only (n = 8) displayed no clinical signs. Clinical signs included refusing sucking, no interest in grouping, neck rigidity, tremors and muscular spasms, ataxia, and hind-limb paralysis. All mice had to be euthanized from 70 to 78 hpi. The supernatant of brain homogenate was used to inoculate C6/36 cells, and CPE was visible at ≈96 hpi for the first passage and at 72 hpi for second and third passages.

The identity of B58 as JEV was confirmed by PCR sequencing. The complete genome sequence of both isolates was then determined. The 2 genomes have identical size at 10,977 nt with a 95-nt 5′ nontranslated region (NTR) and a 583-nt 3′ NTR. The single open reading frame codes for a polyprotein of 3,432 aa. The genomes have similar guanine-cytosine content (51.44% for B58 and 51.39% for GB30).

The 2 bat JEV isolates have an overall sequence identity of 99.9% both at nt and aa levels. When compared to 55 known JEV isolates of known complete genome sequences, the nt sequence identity varies from 88.6% to 99.3%, and aa sequence identity from 97.0% to 99.3%. Analysis of the NTR sequences showed that the bat JEV isolates have the same 5′ NTR as do the others, but the 3′ NTRs of the bat JEVs have a G insertion at nt 307, the same as that observed in the Nakayama strain (GenBank accession no. EF571853).

Phylogenetic trees derived from nucleotide sequences of the complete genome or the most variable envelope protein gene of selected JEV strains (Table 1) indicated that both JEV isolates from bats are members of genotype III as defined by Solomon et al. (*3*). A more detailed analysis indicated that the bat JEVs are most closely related to human isolate LiYujie and mosquito isolate BN19 within cluster 6 (Figure). Similar phylogenetic trees were obtained based on other gene sequences, such as PrM (data not shown).

**Table 1:** Background information of selected strains of Japanese encephalitis virus used in this study*

Strain	Source	Year	Location	GenBank accession no.	Genotype
**B58**	Bat	1986	China	FJ185036	III
**GB30**	Bat	1997	China	FJ185037	III
**Beijing-1**	Human	1949	China	L48916	III
**p3**	Human	1949	China	U47032	III
**Nakayama**	Human	1935	Japan	EF571853	III
**JaOH0566**	Human	1966	Japan	AY508813	III
**Ling**	Human	1965	Taiwan	L78128	III
**ML17-_live**	Human	1981	Taiwan	AY508812	III
**Vellore P20778**	Human	1958	India	AF080251	III
**GP78**	Human	1978	India	AF0723	III
**FU**	Human	1995	Austria	AF217620	II
**WHe**	Pig	NA	China	EF107523	III
**SA14-14-2**	SA-14 derivative	1954	China	AF315119	III
**SA-14**	Mosquito	1954	China	U14163	III
**SH0601**	Mosquito	2006	China	EF543861	III
**JaGAr01**	Mosquito	1959	Japan	AF069076	III
**Ishikawa**	Mosquito	1998	Japan	AB051292	I
**JaOArS982**	Mosquito	1982	Japan	M18370	III
**K87P39**	Mosquito	1987	South Korea	AY585242	III
**K94P05**	Mosquito	1994	South Korea	AF045551	I
**RP-9**	Mosquito	1985	Taiwan	AF014161	III
**CH2195LA**	NA	1994	Taiwan	AF221499	III
BN19	Mosquito	1982	China	FJ185038	III
Liyujie	Human	1979	China	FJ185039	III
YN86-86266	Mosquito	1986	China	DQ404134	I
WTP-70-22	Mosquito	1970	Malaysia	D00998	II
47	Human	1950s	China	AY243810	III
Tla	Human	1971	China	AY243808	III
NACH-13	Human	NA	China	AY243813	III
YNJH04-18	Mosquito	2004	China	DQ404078	III
Chiang Mai	Human	1964	Thailand	U70393	III
G8924	Mosquito	1956	India	EF688636	III
733913	Human	1973	India	AB379813	III
782219	Human	1978	India	EF688655	III
VN118	Mosquito	1979	Vietnam	D00975	III
JKT7003	Mosquito	1981	Indonesia	AY184215	IV

*Boldface indicates strains with complete genome sequences in GenBank. NA, information not available.

**Figure:**
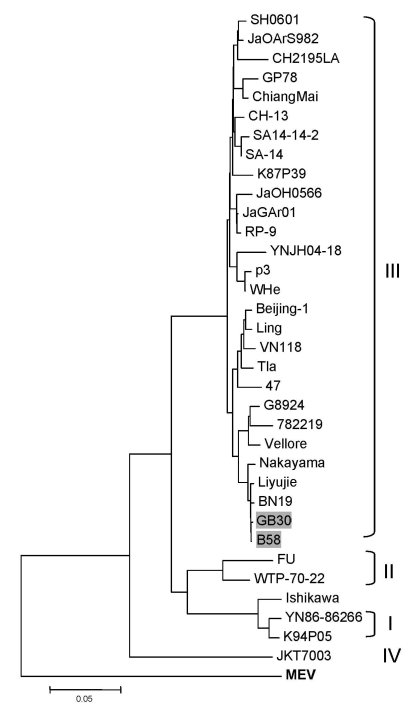
Phylogenetic tree based on the envelope (E) protein gene of selected Japanese encephalitis virus strains. Murray Valley encephalitis virus (MEV) E gene (in **boldface**) was used as an outgroup. Genotypes are indicated on the right. The 2 bat virus isolates used in this study are indicated by shading. Scale bar indicates number of nucleotide substitutions per site. See Table 1 for more details of the strains used in this analysis and their GenBank accession numbers.

Neurovirulence of the 2 bat JEV isolates was determined as described above, and the LD_50_ for suckling mice was 8.0 log_10_/0.02 mL, compared with 3.5 log_10_/0.02 mL for the mosquito strain M10. Neurovirulence of these 2 bat isolates also was predicted from aa sequence comparison to those known to have high neurovirulence (*13**,**14*). As shown in Table 2, all residues important for virulence and neurotropism were conserved between the bat JEV isolates and the Nakayama strain.

**Table 2:** Comparison of key amino acid residues of the E protein of Japanese encephalitis virus important for neurovirulence*

Strain	E107	E138	E176	E177	E264	E279	E315	E439
SA14-14-2	Phe	Lys	Val	Ala	His	Met	Val	Arg
B58	Leu	Glu	Ile	Thr	Gln	Lys	Ile	Lys
GB30	Leu	Glu	Ile	Thr	Gln	Lys	Ile	Lys
Nakayama	Leu	Glu	Ile	Thr	Gln	Lys	Ile	Lys

*These 8 aa residues of the envelope protein are shown to play a key role in neurovirulence. They vary substantially between the attenuated vaccine strain (SA14-14-2) and the virulent strain (Nakayama).

## Conclusions

In this study, we analyzed the complete genome sequences of 2 bat JEV isolates. Although previous serologic studies (*15*) have indicated the occurrence of JEV in a Leschenault’s rousette, we demonstrate JEV infection of *Marina aurata* bats, confirming that the same JEV genotype can infect bats of many species. Both bats are commonly present in Yunnan Province and other parts of China. The bats tend to roost in trees, caves, and roofs of residential properties in close proximity to rice paddocks and pig pens, providing ample opportunity for cross-species transmission among bats and between bats and other animals. Notably, the 2 JEV strains most closely related to the bat viruses were all isolated from Yunnan Province, LiYujie from a human in 1979 and BN19 from mosquitoes in 1982 (Figure).

Our study indicates that the same virus is circulating in hosts of at least 4 different species (human, mosquito, and 2 different bat species), and likely in birds as well, highlighting the broad host range of JEVs in this area. We emphasize that the 4 closely related strains (B58, GB30, LiYujie, and BN19) were isolated over 2 decades, which suggests that the virus was stably maintained in the region, perhaps by circulating in disparate hosts. The findings from our current study highlight the potential importance of bats in human JE outbreaks in the region. Needed additional studies of JEV in bats should include the determination of viremia in bats of different species and potential seasonal variation of viral loads among different bats at different geographic locations. This data would provide a better assessment of risks posed by JEV in bats.

## References

[1] Calisher CH, Childs JE, Field HE, Holmes KV, Schountz T. Bats: important reservoir hosts of emerging viruses. Clin Microbiol Rev. 2006;19:531–45. 10.1128/CMR.00017-06 16847084 PMC1539106

[2] Thiel H-J, Collett MS, Gould EA, Heinz FX, Houghton M, Meyers G, *Flaviviridae.* In: Fauquet CM, Mayo MA, Maniloff J, Desselberger U, Ball LA, editors. Virus taxonomy: the eighth report of the International Committee on Taxonomy of Viruses. San Diego (CA): Elsevier Academic Press; 2005. p. 981–98.

[3] Solomon T, Ni H, Beasley DW, Ekkelenkamp M, Cardosa MJ, Barrett AD. Origin and evolution of Japanese encephalitis virus in southeast Asia. J Virol. 2003;77:3091–8. 10.1128/JVI.77.5.3091-3098.2003 12584335 PMC149749

[4] Wang HY, Takasaki T, Fu SH, Sun XH, Zhang HL, Liang GD, Molecular epidemiological analysis of Japanese encephalitis virus in China. J Gen Virol. 2007; 88:885–94. 10.1099/vir.0.82185-0 17325361

[5] Sulkin SE, Allen R, Sims R. Virus infections in bats. Monogr Virol. 1974;8:1–103. 4367453

[6] Karabatsos N, ed. International catalogue of arboviruses including certain other virus of vertebrates, 3rd ed. San Antonio (TX): American Society for Tropical Medicine and Hygiene; 1985. p. 511–2.

[7] Wang XJ, Zhang YP, Zhang RZ. Analysis on epidemic trend of Japanese B encephalitis during 1998–2002 [in Chinese]. Chinese Journal of Vaccines and Immunization. 2004;10:215–7.

[8] Zhang HL, Zhang YZH, Huang WL. Mi Zhq, Gong HQ, Wang JL. Isolation of Japanese encephalitis virus from brain tissues of bat in Yunnan Province [in Chinese]. Virol Sin. 2001;16:74–7.

[9] Wang JL, Zhang HL, Zhou JH, Liang GD. Genotyping of Japanese encephalitis viruses isolated in Yunnan [in Chinese]. Zhonghua Shi Yan He Lin Chuang Bing Du Xue Za Zhi. 2008;22:87–90. 18574523

[10] Reed LJ, Muench H. A simple method of estimating fifty percent endpoints. Am J Hyg. 1938;27:493–7.

[11] Kuno G, Chang GJ, Tsuchiya KR, Karabatsos N, Cropp CB. Phylogeny of the genus *Flavivirus*. J Virol. 1998;72:7383. 10.1128/jvi.72.1.73-83.1998 PMC109351 9420202

[12] Kumar S, Tamura K, Nei M. MEGA: molecular evolutionary genetics analysis software for microcomputers. Comput Appl Biosci. 1994;10:189–91. 10.1093/bioinformatics/10.2.189 8019868

[13] Trent DW, Barrett AD, Ni H, Chang GJ, Xie H. Molecular basis of attenuation of neurovirulence of wild-type Japanese encephalitis virus strain SA14. J Gen Virol. 1995;76:409–13. 10.1099/0022-1317-76-2-409 7844560

[14] Cecilia D, Gould EA. Nucleotide changes responsible for loss of neuroinvasiveness in Japanese encephalitis virus neutralization-resistant mutants. Virology. 1991;181:70–7. 10.1016/0042-6822(91)90471-M 1704661

[15] Cui J, Counor D, Shen D, Sun G, He H, Deubel V, Detection of Japanese encephalitis virus antibodies in bats in southern China. Am J Trop Med Hyg. 2008;78:1007–11. 18541785

